# Impact of foliar spray with Se, nano-Se and sodium sulfate on growth, yield and metabolic activities of red kidney bean

**DOI:** 10.1038/s41598-023-43677-8

**Published:** 2023-10-10

**Authors:** Nada Abouelhamd, Fatma Abd El Lateef Gharib, A. A. Amin, Eman Zakaria Ahmed

**Affiliations:** 1 Department of Botany and Microbiology, Faculty of Science, Helwan University, Cairo, Egypt; 2 Department of Botany, National Research Centre, Dokki, Cairo, 12622 Egypt

**Keywords:** Physiology, Plant sciences

## Abstract

Sulfur (S) is an essential microelement for plants. Based on the chemical similarity between Se and S, selenium may affects sulphur uptake by plants. This work aimed at investigating the effect of foliar spray with sodium selenate, gum arabic coated selenium nanoparticles (GA-SeNPs ≈ 48.22 nm) and sodium sulfate on red kidney bean (*Phaseolus vulgaris* L.) plants. Each treatment was used at 0.0, 1, 5, 10 and 50 µM, alone or combination of sodium sulfate with either Se or nano-Se, each at 0.5, 2.5 and 5 µM concentrations. The effect of foliar spray on vegetative growth, seed quality, and some metabolic constituents of red kidney bean (*Phaseolus vulgaris* L.) plants were investigated. Selenium nanoparticles have been synthesized through the green route using gum arabic (as a stabilizing and coating agent. Foliar application of different concentrations of Se, nano-Se, Na_2_SO_4_ up to 10 μM and their interaction were effective in increasing the growth criteria (i.e. shoot and root lengths, plant fresh and dry weights, number of leaves and photosynthetic area (cm^2^ plant^−1^).There was also a significant increase in photosynthetic pigment contents, yield (i.e., 100-seed weight), total carbohydrate, crude proteins and mineral contents in both leaf as compared to their untreated control plants. Furthermore, interaction between sodium sulfate with nano-Se or Se, each at 5 µM significantly increased the vegetative growth, 100-seed weight, and pigment contents in leaves and improved the nutritional value and quality of red kidney bean seeds.

## Introduction

Common bean (*Phaseolus vulgaris* L.) is a dietary protein, amino acid, fiber, complex carbohydrate, vitamin, mineral, phenol and antioxidant compound^1,2^. Common bean is an important cereal legume that is consumed worldwide for edible seeds and pods. It is the third most important legume after soybean and peanut^3,4^. Regionally, Asia was recorded the first in dry bean production with about 43% of global production, followed by the Americas North, Central, and South America (29%), and Africa (26%). Europe and Oceania contribute about 2% of total production^5^. Harvest residues such as dried pods, straw and processing by-products can be used as animal feed^6^. Red bean varieties contain low fat, high levels of protein, and some bioactive compounds^7^. Red kidney beans are best source of vitamin B group, essential minerals like K, Ca, Mg, P and Fe compared to other varieties^8,9^. However, nutritional value is limited by low concentrations of the essential sulfur-containing amino acids methionine (Met) and cysteine (Cys)^10^. Except for cysteine, methionine and tryptophan, the raw and processed red kidney beans replenish the FAO/WHO amino acid needs for teenagers^11^. Plenty of studies are focusing to improve sulfur-containing amino acids crops through transgenic development^12^, synthetic protein synthesis^13^, or traditional breeding^14^. Improvement of Sulphur role in plants can be studied by traditional ways like sulphur supplement in suitable forms and doses but we can also focus on other elements which are transported by the same routes and it’s metabolism in plant is similar to sulphur like selenium.

Selenium (Se) is a trace element that has been regarded as an essential element for both human and animals. Selenium is a structural component of several enzymes with physiologically antioxidant properties including glutathione peroxidases and thioredoxin reductase so it is associated with the enhancement of antioxidant activity in plants, animals and humans^15,16^. Selenium is not an essential nutrient for plants but plays important roles in alleviating the abiotic stresses suffered by plants^17^. Nanoparticles of elemental selenium (Se^0^) have unique physical and chemical properties, which differ from the properties of the corresponding bulk materials^18^. Se-nanoparticles could be synthesized within the reduction of a Se-salt, usually in the presence of a stabilizing agent to prevent the clusters of Se atoms from growing^19^. It is important to study sulfur relation and interaction with selenium, as selenium shares the same chemical properties with sulfur, therefore it is taken up inside the plants via sulfate transporters and assimilated by the sulfur assimilating pathway^20– 22^, so it is important to study its relation and interaction with selenium.

Sulfur (S) is known to be one of the most crucial major nutrients essential for plant growth^23^. Sulfur, the fourth essential macronutrient after nitrogen (N), phosphorus (P), and potassium (K) in plants. Once sulfate is taken up to plant cells by sulfate transporters (SULTR). Sulfur is readily assimilated into sulfur-containing essential amino acids (cysteine and methionine) as well as the antioxidant glutathione (GSH)^24^. This Cys is finally channelized into S-containing metabolites e.g. GSH, and metal-chelating proteins—Phytochelatins (PCs) and metallothioneins (MTs), which play pivotal roles in heavy metal stress tolerance mechanisms. The excess sulfur transported to shoots is transiently accumulated in the vacuoles and serves as a large S reserve for plant metabolic activities^25^.

Recently, more and more attention has been paid to the nutritional and pharmaceutical values of Se and sulfur-containing plant products, which are used as important tools for improving crop growth and productivity. It delays^26^, increases resistance to oxidative stress^27^, and improves plant resistance^28^. At low doses, Se significantly increased plant growth and rapeseed yield components^29^, wheat^30^ and cowpea plants^31^, whereas, at high doses, Se acts as a prooxidant and catalyzes the oxidation of thiols and generates superoxide that can damage cellular components^32^, resulting in metabolic disturbances and a reduction in cucumber yield^33^. NanoSe significantly stimulated tobacco organogenesis and root system growth^34^ and improved grape bean growth and yield^35^. Low concentrations of NanoSe and Se improved the growth parameters and chlorophyll content of tomato plants under temperature stress^36^ and sage plants under normal and salt stress^37^. Sulfur is required throughout the plant growth, from vegetative to harvesting^38^ and plays vital roles in the activation of reactive oxygen species (ROS) scavenging enzymes to improve antioxidant defense under abiotic stresses^39^. Indirectly, S interacts with other molecules, e.g., auxins, cytokinin, gibberellins, ethylene, jasmonic acid, and salicylic acid, to counteract abiotic stresses^40^. Plants need thiol-containing S biomolecules to develop a defensive mechanism against different abiotic stresses^41^. Fertilization with sulfur is required in agricultural areas, since plants readily uptake sulfur from the soil predominantly in sulfate (SO_4_^−2^). Sulfate is generally available in very low amount in the soil, as it is water soluble, therefore it quickly loses from the soil by leaching^42^. The efficacy of S to modulate plant physiology depends on its concentration, application method and plant genetics^43^. Studies on improving plant growth are continuously updated worldwide aiming to find more suitable and inexpensive treatments with stimulatory effects. This work aimed to synthesis Se nanoparticles, evaluate the morphological and physiological response of red kidney bean (*Phaseolus vulgaris* L.) plants to foliar application of sodium selenate, gum arabic-coated nano selenium and sodium sulfate, in addition to the interactions between sodium sulfate (sulfur) with either selenium or nano-Se. The outcome of this work may be useful to determine the possibility of using the most suitable concentrations for further field agricultural applications on red kidney bean plants.

## Materials and methods

### Plant materials

A pure lot of red kidney bean (*Phaseolus vulgaris* L.) seeds were provided by Sakha Horticulture Research Station, Horticulture Research Institute, Agricultural Research Center, Egypt.

### Chemicals

All chemicals used in this study were of high purity, purchased from Sigma-Aldrich Chemical Co., Germany and Merck (Rio de Janeiro, RJ, Brazil). Distilled and deionized water was used in all experimental work.

### Preparation of gum arabic-coated selenium nanoparticles (GA-SeNPs)

**Figure 1:**
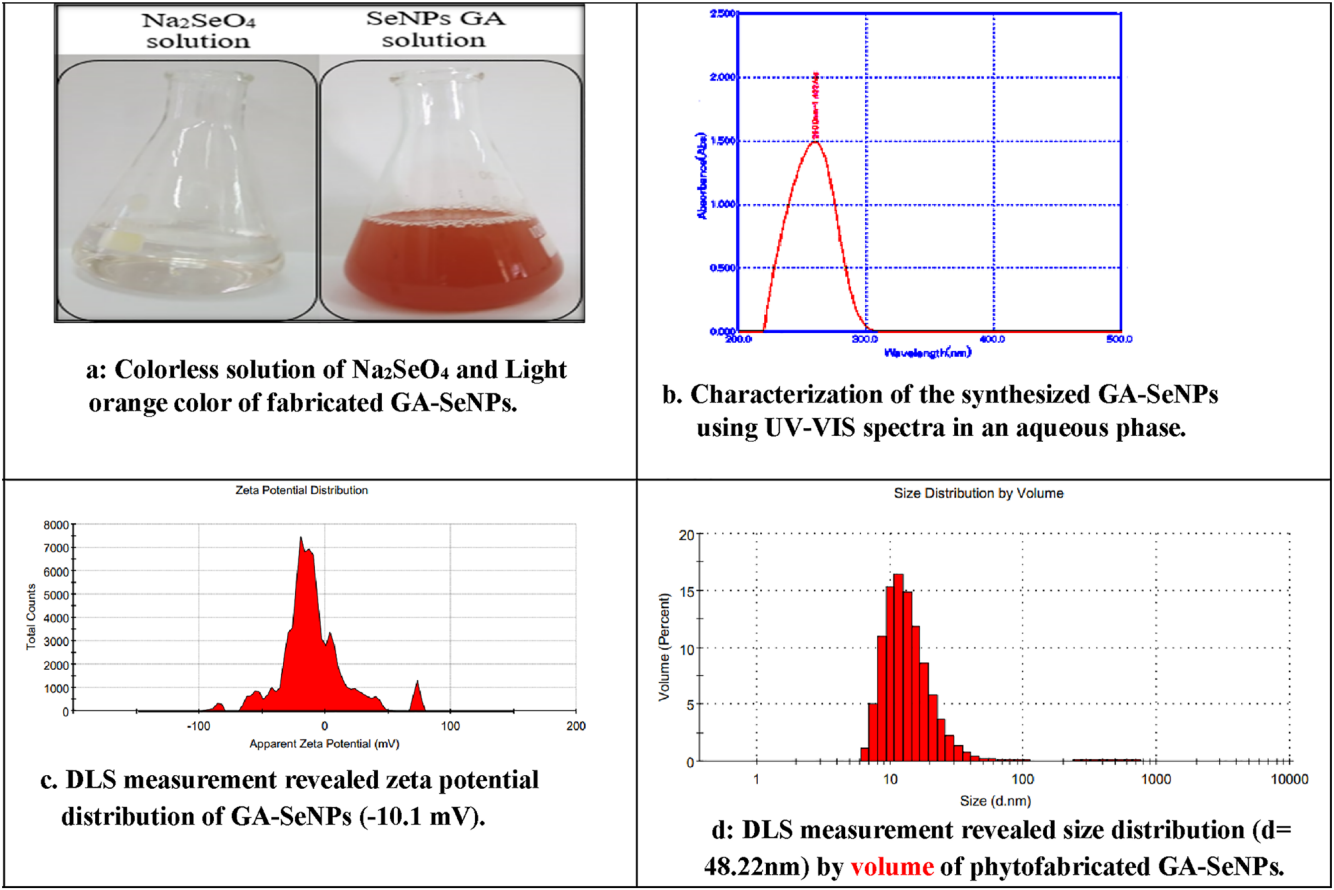
(**a**) Colorless solution of Na_2_SeO_4_ and Light orange color of fabricated GA-SeNPs. (**b**) Characterization of the synthesized GA-SeNPs using UV–Vis spectra in an aqueous phase. (**c**) DLS measurement revealed zeta potential distribution of GA-SeNPs (− 10.1 mV). (**d**) DLS measurement revealed size distribution (d = 48.22 nm) by volume of phytofabricated GA-SeNPs.

Gum arabic-coated selenium nanoparticles (GA-SeNPs ≈ 48.22 nm) was synthesized by the reduction of sodium selenate, using ascorbic acid according to Malhotra et al.^44^ method with slight modification. A stock of aq. solution of 10 mM sodium selenate^45^ and ascorbic acid powder 1.5% (w/v) were allowed to react with each other until color changed from colorless to light orange. Gum Arabic solution (10%) was added with continuous stirring to the previously prepared nano selenium solution (Fig. 1a), then different concentrations were prepared using deionized water.

### Characterization of selenium nanoparticles

#### UV–Visible spectrophotometer (UV–Vis)

Absorbance of nanoparticles was performed by ACCULAB Spectrophotometer, model UVS-26OD, made in USA, SN:UVS-2501712053 at Central Laboratory-Faculty of Science, Helwan University. Spectra of GA-SeNPs solution was recorded as a function of wavelength in the range of 200–500 nm at a resolution of 1 nm.

#### Dynamic light scattering (DLS) and zeta-potential analysis

DLS measurements were performed at Central Laboratory, Faculty of Science, Helwan University using Zetasizer Nano ZS particle analyzer (Malvern Instruments, Malvern Ltd) in order to determine the average particle size, size distribution and zeta potential of GA-SeNPs at 25 °C. The DLS measurements were performed under the following conditions: Dispersant dielectric constant 78.5, material refractive index 1.30, dispersant (water), dispersant RI 1.33, viscosity 0.8872 cP, count rate 306.2 kcps, measurement position 5.50 mm, material absorption 0.001 and using clear disposable zeta cell.

#### Transmission electron microscopy (TEM)

Transmission electron microscopy characterization by (TEM) JEM-2100 HR was carried out at Central Labs., Faculty of Agriculture, Cairo University, Cairo, Egypt. TEM studies were prepared by dropping selenium nanoparticles onto carbon-coated TEM grids. The Film on the TEM grids was allowed to dry and the extra solution was removed using a blotting paper.

#### Fourier transform infrared spectrometer (FT-IR)

FT-IR measurements were carried out in order to obtain information about transformation of functional groups due to the reduction process and chemical groups present around SeNPs as a coat for their stabilization. The measurements were carried out using Perkin Elmer Spectrum two infra-red spectrometer at Central Laboratory, Faculty of Science, Helwan University.

### Experimental design

A pot experiment was conducted at the Experimental farm of Helwan University, on 4th August 2019. A homogenous lot of red kidney bean seeds were sown in clay pots, 40 cm in diameter and filled with 15 kg of clay loamy soil (consisting of 50.04% clay, 28.96% silt, 15.86% fine sand and 5.14% coarse sand). The well-established seedlings were thinned to 5 plants per pot after 15 days from sowing.

The experiment consisted of 19 treatments (three metals (Se, GA-SeNPs and S)—four concentrations (1, 5, 10 and 50 µM)—six interaction treatments and the negative control) with six replicates and five plants per replicate; therefore, 12 plants were used for growth measurement in each treatment. The pots were arranged in complete randomized blocks design with 19 treatments.

The pots were regularly irrigated with tap water to keep the moisture content of the soil to 70% field capacity. Tap water was used for irrigation after making sure by analysis of water samples that there is no selenium detected in water.

Fertilization was carried out for each pot at the proportion of 1 g ammonium nitrate (33.5% N), 2 g calcium superphosphate (15.5% P_2_O_5_) and 1 g potassium sulfate (48% K_2_SO_4_). These fertilizers were applied in two doses after sowing.

### Spraying treatments

A foliar spray was applied twice to red kidney bean plants during the vegetative stage (at 24 and 31 days after sowing (DAS)), with 500 mL of one of the following freshly prepared solutions.

#### The control group: foliar sprayed with distilled water

The Na_2_SeO_4_, GA-SeNPs and Na_2_SO_4_ groups: each subdivided into four subgroups and foliar sprayed with Na_2_SeO_4_, GA-SeNPs or Na_2_SO_4_ solutions each at 1, 5, 10 and 50 µM concentrations.

The Na_2_SeO_4_ + Na_2_SO_4_ or GA-SeNPs + Na_2_SO_4_ groups: each subdivided into three subgroups and foliar sprayed with Na_2_SeO_4_ + Na_2_SO_4_ or GA-SeNPs + Na_2_SO_4_ solutions, each at 0.5, 2.5 and 5 µM concentrations.

The volume of the spraying solution was maintained just to cover completely the plant’s foliage till dripping.

**Figure:**
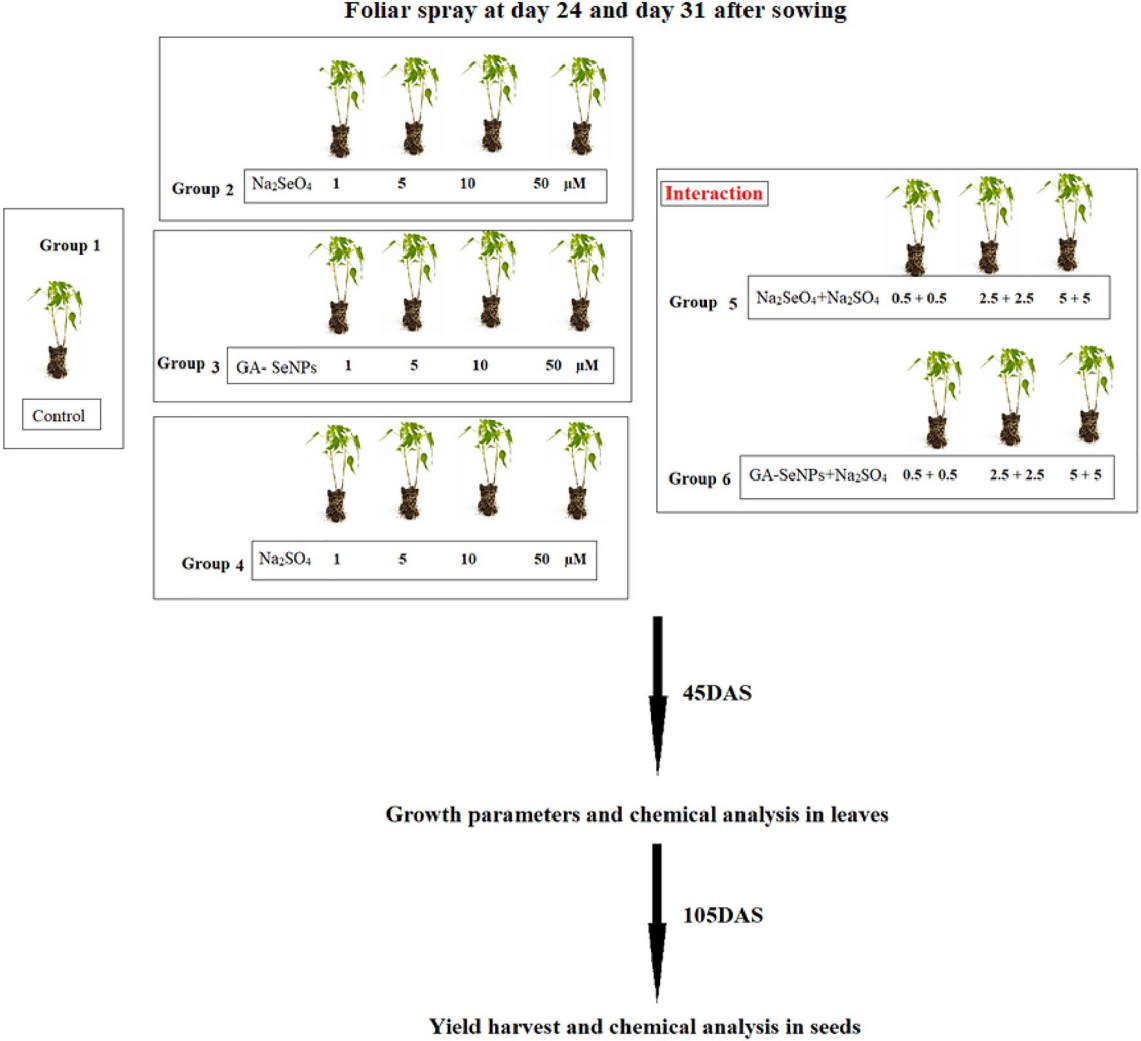
Graphical scheme illustrating the experiment design.

### Sampling

At pre flowing stage (45 DAS), twelve plants (six replicates) were randomly harvested. From each of the experimental groups. Different growth parameters (i.e., length of stem and root (cm), leaf numbers, fresh weights (FW) and dry weights (DW) of shoot and root (g plant^−1^), and total leaf area (cm^2^ plant^−1^) were calculated according to Koller^46^. Dry weights were obtained by drying plant samples in an oven with a drift fan at 70 °C until constant weights and minerals content were determined in leaves. Fresh leaves samples were taken from each treatment for determination of photosynthetic pigments.

The seeds of each treatment were harvested at the fruiting stage (105 DAS). The dried seeds were used for determination of 100 seed weight (g plant^−1^), total carbohydrates, crude protein and minerals chemical analysis.

### Determination of photosynthetic pigments

Photosynthetic pigments (mg g^−1^ FW) in fresh kidney bean leaves were measured according to the method achieved by Metzener et al.^47^.

### Total carbohydrates

The total carbohydrates (TC) was determined in dry powdered samples of leaves and seeds using anthrone technique according to Umbriet et al.^48^.

### Crude protein

Crude protein percentage (CP) in the dry samples of leaf and seeds was calculated by multiplying the values of total N by 6.25^49^.

### Determination of minerals

Mineral ion content in air-dry leaves and seeds of red kidney bean developed from different treatments were estimated in the Ecology Laboratory, Faculty of Science, Helwan University using Microwave Plasma Atomic Emission Spectroscopy (Agilent Technologies 4210 MP-AES). The instrumental settings and operational procedures were adjusted according to the Manufacturer’s User Manual.

The nitrogen content in the leaf and seed was determined by the modified Micro-Kjeldahl method according to AOAC^49^.

### Statistical analysis

The data were expressed as the average of 6 replicates for growth criteria and as the average of 3 of all chemical analyzes. One-way ANOVA (LSD. And Duncan’s multiple comparison test) at 0.05 were performed using IBM SPSS Statistics for Windows 21 software.

### Ethical approval and consent to participate

Samples were provided by Sakha Horticulture Research Station, Horticulture Research Institute, Agricultural Research Center, Egypt. But no herbarium voucher specimen of this plant has been deposited in a publicly available herbarium as it is cultivated not wild plant. Permission was provided from the governate as a researchers form Helwan University. Experimental research and field studies on plants comply with relevant institutional, national, and international guidelines and legislation.

## Results

### Color of the synthesized GA-SeNPs

Figure 1a shows the color of the synthesized GA-SeNPs at 10 mM sodium selenate, 1.5% ascorbic acid and 10% gum arabic at pH 2.6. The appearance of light orange color, specific for SeNPs indicates the fabricated GA-SeNPs, as a result of reduction of selenium ions into selenium nanoparticles by ascorbic acid. The color change is due to the surface plasmon resonance phenomenon (SPR).

### UV–Visible spectrophotometer (UV–Vis)

Spectra of GA-SeNPs solution was recorded as a function of wavelength in the range of 200–500 nm at a resolution of 1 nm (Fig. 1b). A strong absorption peak was observed between 230 and 310 nm with maximum absorbance at 260 nm, which is characteristic for colloidal nano-selenium. Previous studies have shown that the spherical Se-NPs contribute to the absorption bands at around 250–400 nm in the UV–Visible spectra. Shah et al.^50^ reported λ max at 270 nm and Gharib et al.^31^ at 296 nm. The broad obvious peak at 260 nm demonstrates that the reducing agent was strong enough to ensure the complete conversion of the precursor molecules into nano-sized selenium particles.

### Dynamic light scattering (DLS)

The DLS measurement of the biosynthesized GA-SeNPs indicates that the size distribution by volume is registered around 48.22 nm and PdI = 0.341 (Fig. 1c,d), which is in agreement with TEM (≈ 46.9) images. Of course, slight differences between these two techniques arise because TEM images were recorded from a limited area (30 μm × 30 μm) of a dried film of GA-SeNPs, meanwhile the DLS measurements were performed in solution. Furthermore, the apparent zeta potential was recorded at a maximum value of − 10.1 mV, which indicates that these nanoparticles do not form aggregates in solution leading to a stable dispersion. Similarly, the DLS method revealed SeNPs particles with maximum distribution registered around 400 nm and an apparent zeta potential value of − 14.2 mV^51^.

### Fourier transform infrared spectrometer (FT-IR)

**Figure 2:**
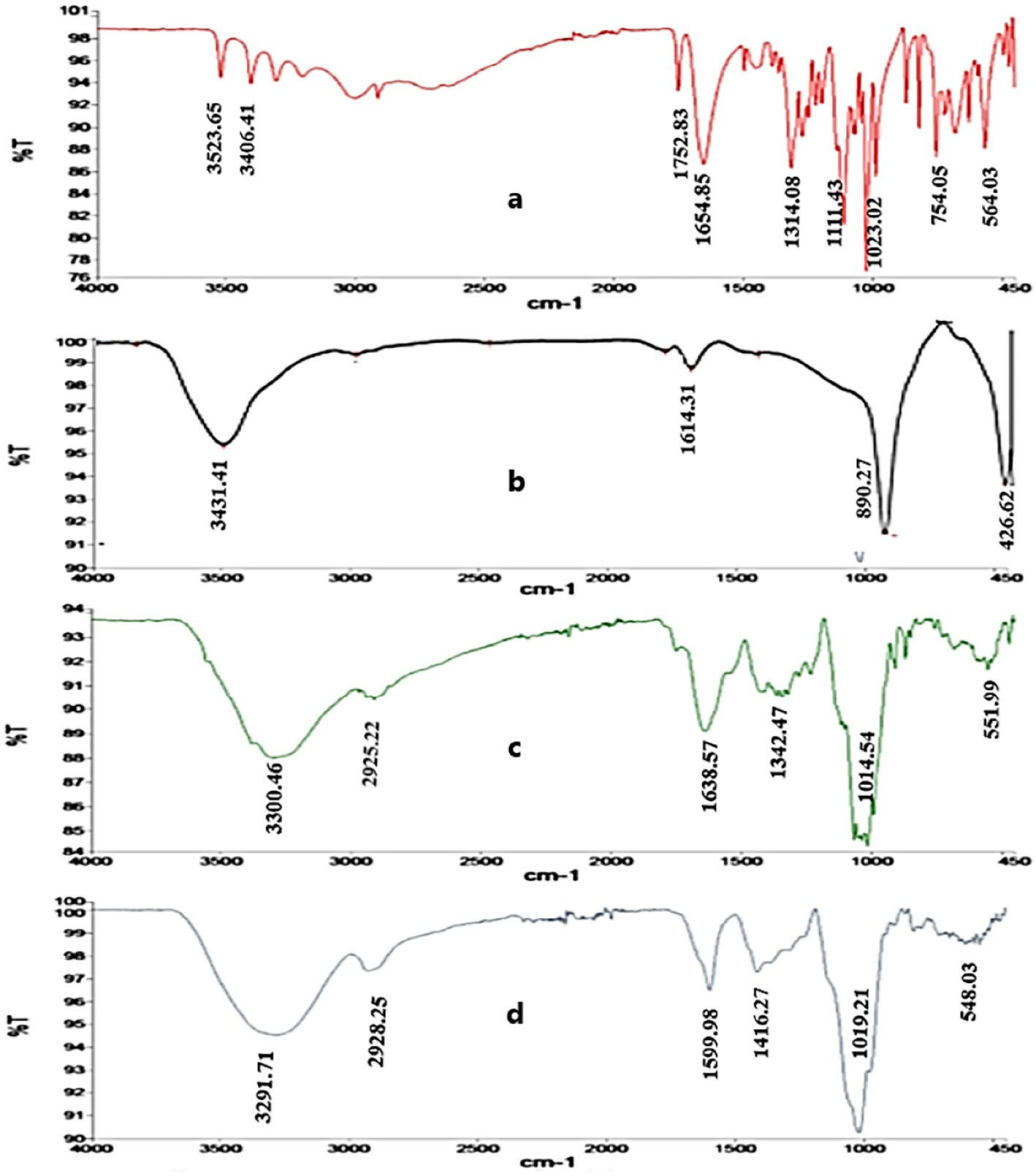
FTIR spectra of (**a**) ascorbic acid, (**b**) Na_2_SeO_4_, (**c**) gum Arabic and (**d**) gum Arabic-capped-SeNPs, formed at 10 mM sodium selenate and 1.5% ascorbic acid.

The FT-IR spectrum of pure L-Ascorbic acid (Fig. 2a), sodium selenate (Fig. 2b) and gum acacia powder (Fig. 2c) were closely matched with the reported literature.

The FT-IR spectrum of gum Arabic-coated selenium nanoparticles (GA-SeNPs) (Fig. 2d) show an intense absorption peaks at 3291.71 cm^−1^, 2928.25 cm^−1^, 1599.98 cm^−1^, 1416.27 cm^−1^, large intense band at 1019.21 cm^−1^ and at 548.03 cm^−1^ associated with -OH stretching of the aromatic rings, ether-methoxy-OCH_3_ groups, amide I (C=O stretch of the ester group), (C–H asymmetric bending in CH_2_ and CH_3_ groups), the superposition of in-plane C–H bending and the characteristic Se–O stretching vibration and the –COOH group/–OH of GA-SeNPs were shifted to different wave numbers than that of pure GA (3300.46 cm^−1^, 2925.22 cm^−1^, 1638.57 cm^−1^, 1342.47 cm^−1^, 1014.54 cm^−1^ and 551.99 cm^−1^), respectively. In accordance with the present results, previous studies have observed peaks at 3280 cm^−1^ and 2918 cm^−1^ in FTIR spectrum indicate the presence of a biopolymer associated with the SeNPs obtained from parsley extract^51^, a shift in peak from 1417 to 1384 cm^−1^ indicating H–C–OH bond in dextrin coated selenium nanoparticles^35^ and shift in peak from 551 to 559 cm^−1^ supporting the bridging followed by linkage involving the Ag surface and oxygen atoms of carboxyl groups in the gum Arabic-capped-AgNPs^52^. The shift of the OH band indicates a strong bonding interaction between hydroxyl groups of gum Arabic and surface atoms of SeNPs, which plays an important role in the stabilization of nanoparticles^53^. The results of the FTIR spectra of gum Arabic and gum Arabic-capped Se nanoparticles confirm the bonding between SeNPs with –OH/COO– groups, thereby stabilizing the nanoparticles formed.

### Transmission electron microscopy (TEM)

**Figure 3:**
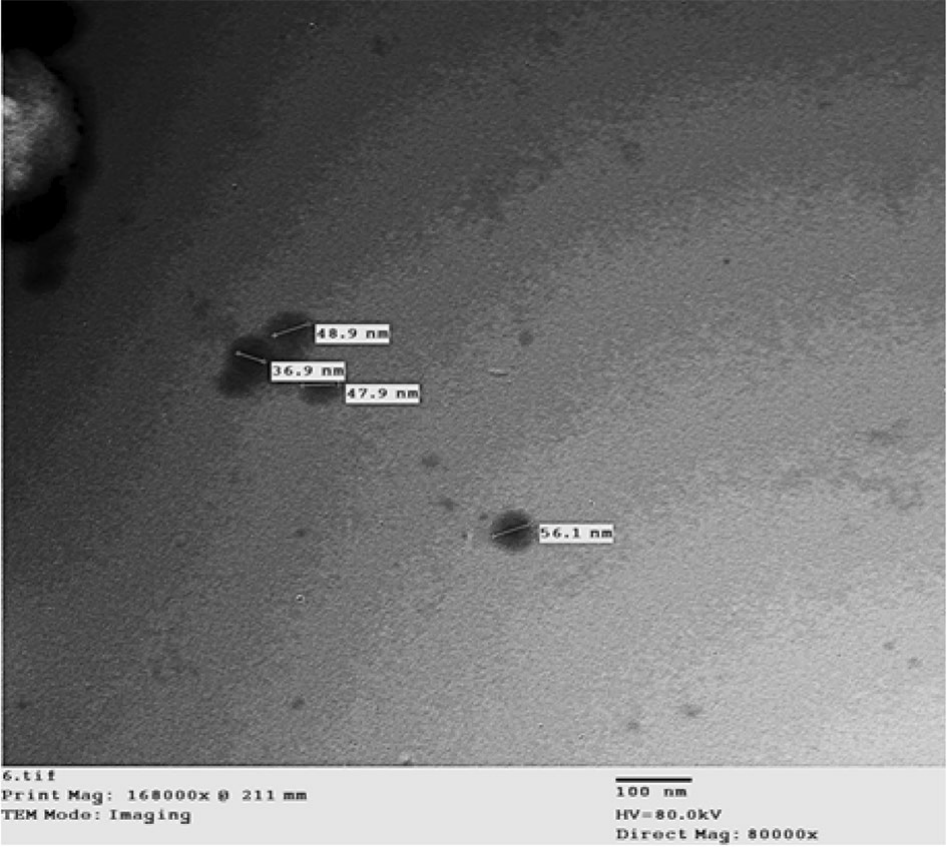
TEM micrograph of spherical shaped synthesized SeNPs performed at 10 mM sodium selenate, 1.5% ascorbic acid and coated with gum Arabic (≈ 46.9 nm, including coat).

Figure 3 shows a TEM micrograph of spherical shape synthesized gum arabic-coated selenium nanoparticles (GA-SeNPs_)_, with a mean particle size diameter of 46.9 nm.

### Growth parameters

**Figure 4:**
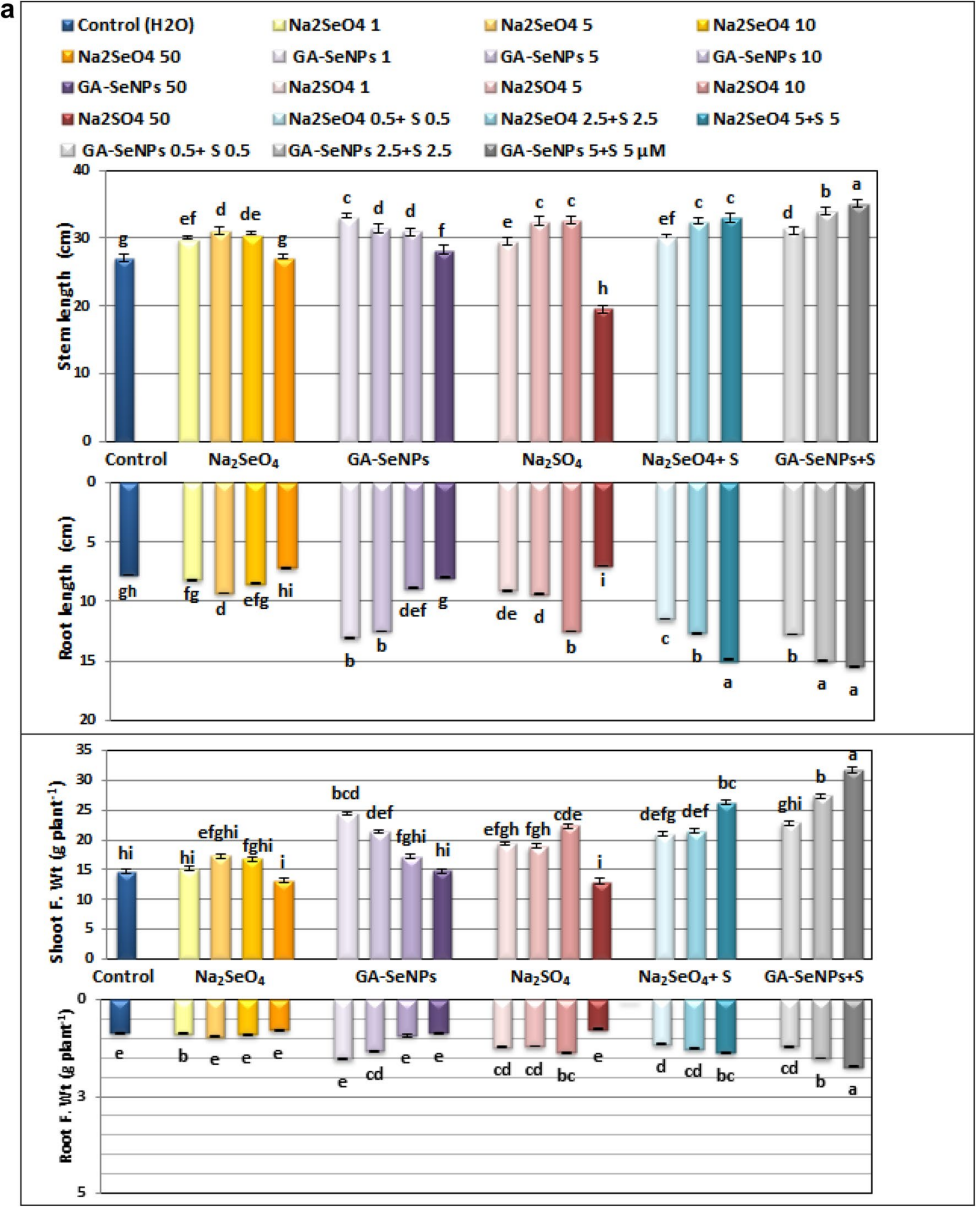

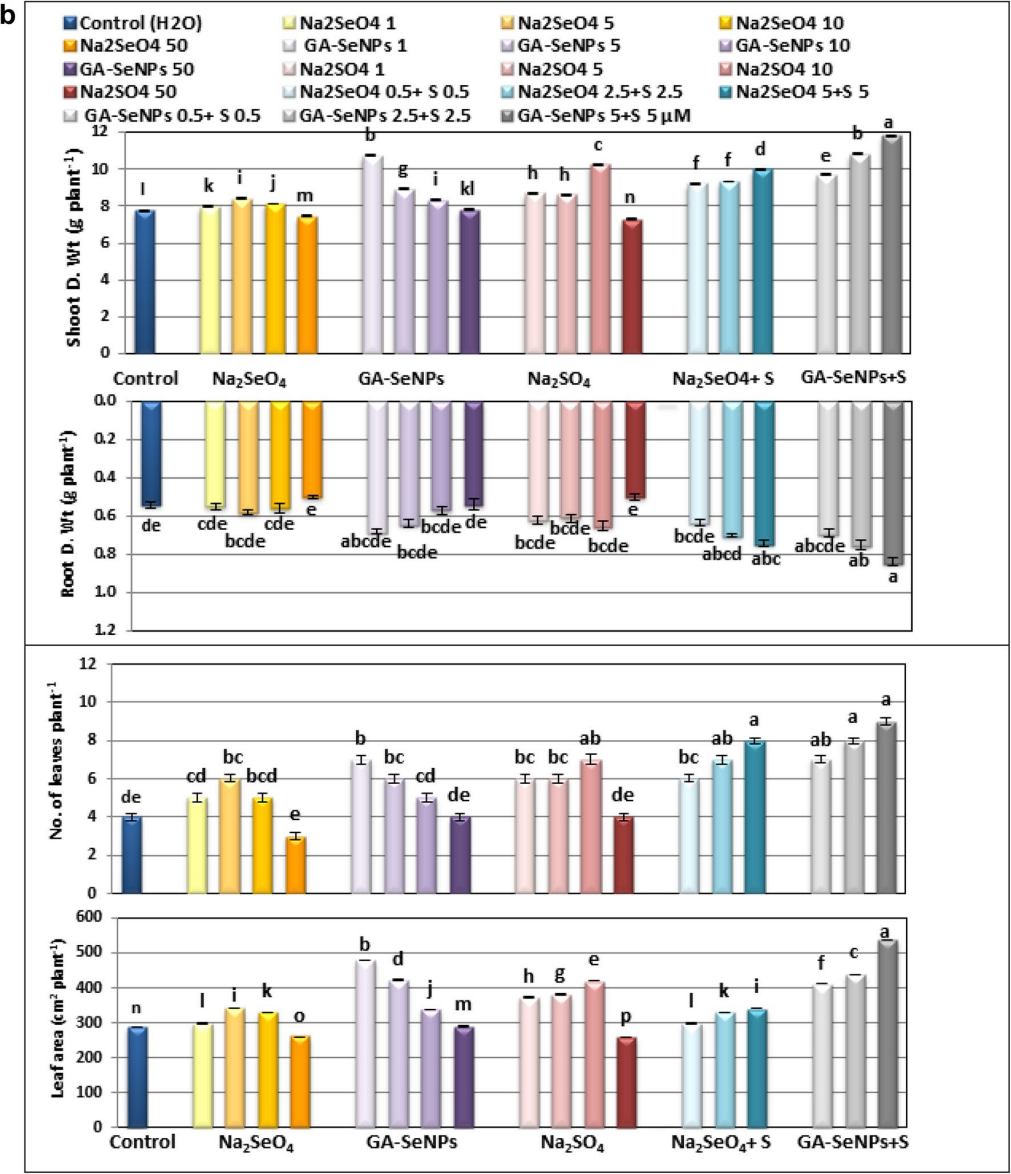
(**a**) Effect of foliar spray with sodium selenate (Na_2_SeO_4_), selenium nanoparticles (GA-SeNPs), and sodium sulphate (Na_2_SO_4_), each at 0.0, 1, 5, 10, and 50 µM and interaction between sodium sulphate with either sodium selenate or selenium nanoparticles, each at 0.5, 2.5 and 5 µM on growth characteristics of red kidney bean (*Phaseolus vulgaris* L.) plants at 75 days from sowing. Each result is a mean of 6 replicates. Statistical analysis was carried out using Duncan test. Different letters show significant variation at 0.05 P. Vertical bars represent ± SE. (**b**) Effect of foliar spray with sodium selenate (Na_2_SeO_4_), selenium nanoparticles (GA-SeNPs), and sodium sulphate (Na_2_SO_4_), each at 0.0, 1, 5, 10, and 50 µM and interaction between sodium sulphate with either sodium selenate or selenium nanoparticles, each at 0.5, 2.5 and 5 µM on growth characteristics of red kidney bean (*Phaseolus vulgaris* L.) plants at 75 days from sowing. Each result is a mean of 6 replicates. Statistical analysis was carried out using Duncan test. Different letters show significant variation at 0.05 P. Vertical bars represent ± SE.

**Figure 5:**
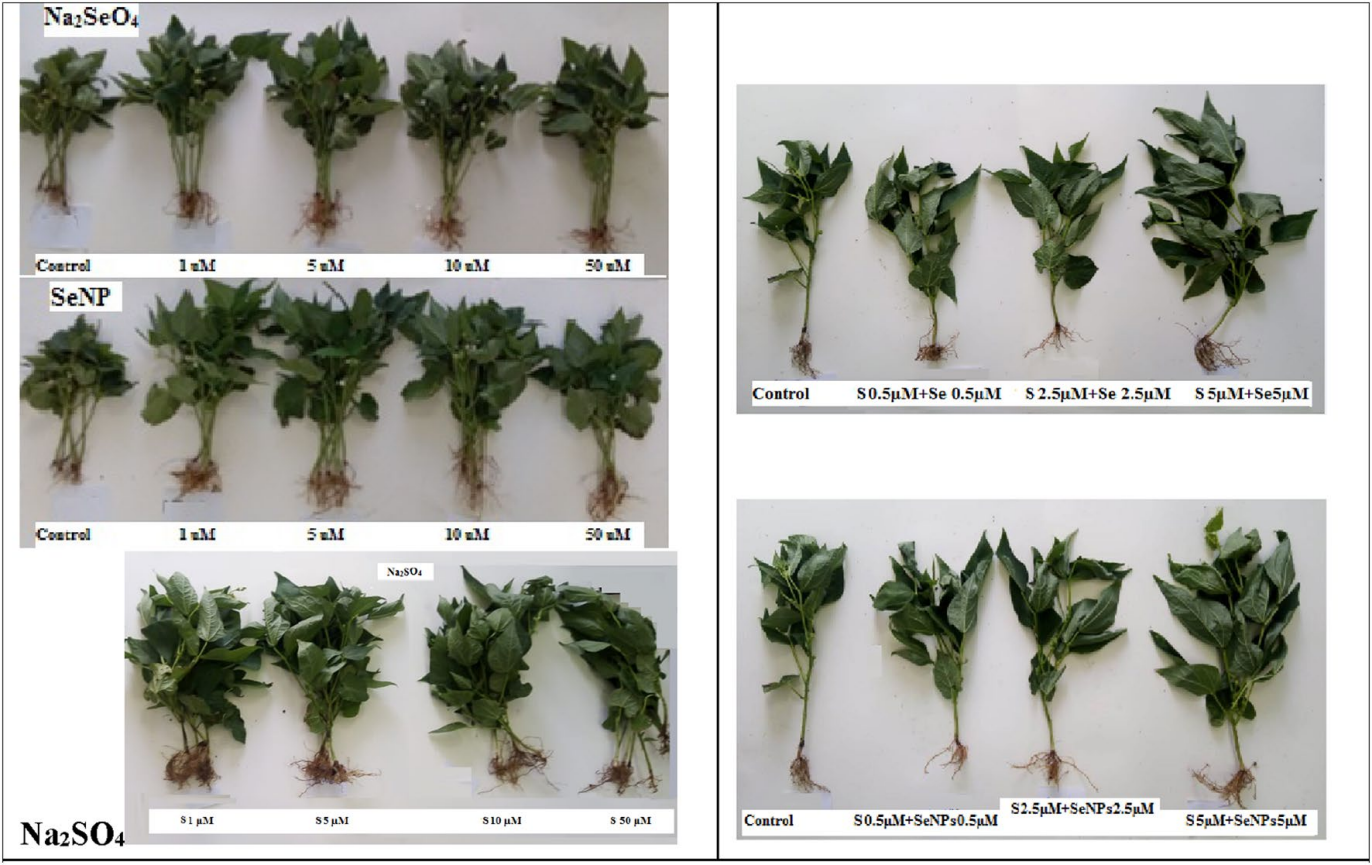
Growth of Red kidney beans (*Phaseolus vulgaris* L.) plants at 45 days from sowing as affected by foliar spray with sodium selenate (Na_2_SeO_4_), selenate nanoparticles (GA-SeNPs) and sodium sulphate (Na_2_SO_4_), each at (0.0, 1, 5, 10 and 50 µM). Each group consists of 5 plants, and interaction between Na_2_SO_4_ with either Na_2_SeO_4_ or GA-SeNPs, each at 0.5, 2.5 and 5 µM, each group consists of one plant.

The data presented in Figs. 4a,b and 5 revealed that foliar application of Na_2_SeO_4_, GA-SeNPs or Na_2_SO_4_, up to 10 µM as well as the combination of sodium sulfate with either selenium or nano-Se at 0.5, 2.5 and 5 µM significantly promoted all measured growth criteria (lengths of stem and root, fresh and dry weights of the shoot and root, No. of leaves and total leaves area plant) of red kidney bean plants at 45 DAS. At low concentrations the increment in growth parameters was often highly significant compared to the corresponding untreated control plants.

At 50 µM GA-SeNPs increased only stem length while there was no significant increase in other measured parameters. While in case of Na_2_SeO_4_ and Na_2_SO_4_ the highest concentration 50 µM markedly decreased most growth parameters in comparison with untreated controls plants. The most effective treatments on growth parameters were obtained with SeNPs at 1 and 5 µM, followed by Na_2_SO_4_ at 10 µM, then Na_2_SeO_4_ at 5 µM concentration.

Furthermore, a combination of nano selenium + sodium sulfate each at 0.5, 2.5 and 5 µM significantly increased the growth criteria of red kidney bean plants more than the combination of selenium + sodium sulfate at the same concentrations. The highest recorded values in stem and root lengths (35.13 and 15.50 cm), fresh and dry weights of the shoot (31.78 and 11.81 g plant^−1^) and root (1.73 and 0.84 g plant^−1^), No. of leaves (9) and total leaves area (328.92 cm^−2^ plant^−1^) were obtained with GA-SeNPs at 5 µM + Na_2_SO_4_ at 5 µM concentration, compared to (27.08 and 7.77cm, 14.69, 7.74, 0.86 and 0.54 g plant^−1^, 4 No. and 288.29 cm^−2^ plant^−1^), respectively for corresponding untreated control plants (Figs. 4a,b, 5).

### Yield (quality of yielded seeds)

**Figure 6:**
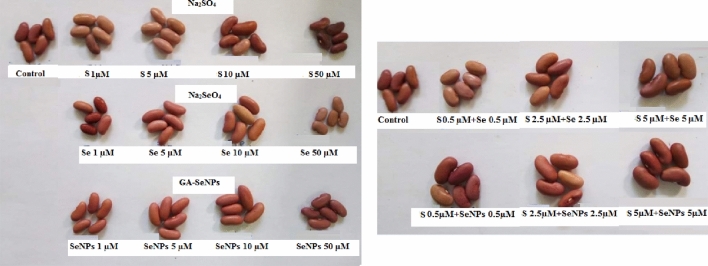
Seeds of red kidney bean (*Phaseolus vulgaris* L.) plants at 105 days from sowing as affected by foliar spray with sodium selenate (Na_2_SeO_4_), selenate nanoparticles (GA-SeNPs) and sodium sulphate (Na_2_SO_4_), each at (0.0, 1, 5, 10 and 50 µM) and interaction between sodium sulphate with either sodium selenate or selenium nanoparticles, each at 0.5, 2.5 and 5 µM, each group consists of 5 seeds.

**Figure 7:**
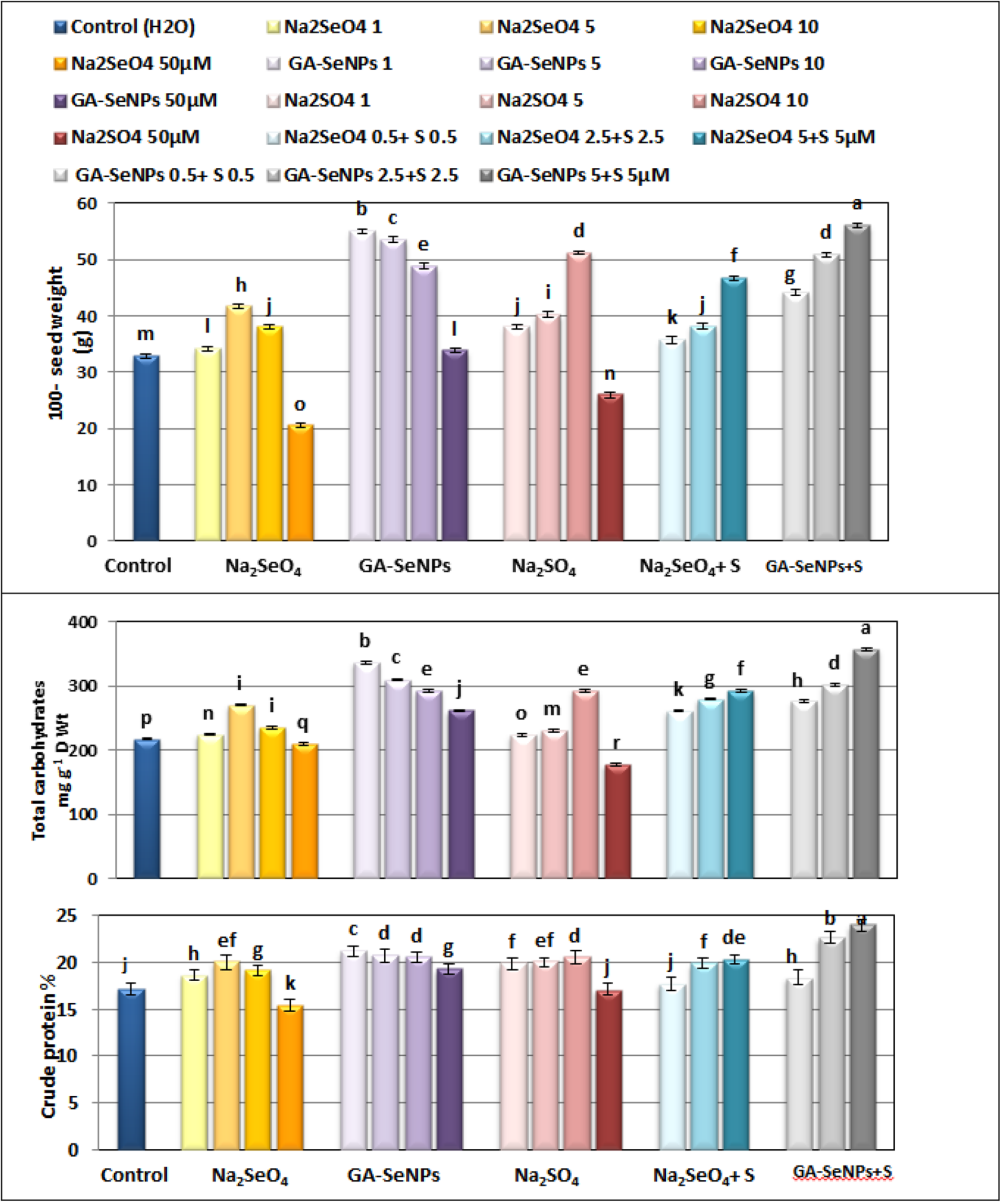
Effect of foliar spray with sodium selenate (Na_2_SeO_4_), selenium nanoparticles (GA-SeNPs), and sodium sulphate (Na_2_SO_4_), each at 0.0, 1, 5, 10, and 50 µM and interaction between sodium sulphate with either sodium selenate or selenium nanoparticles, each at 0.5, 2.5 and 5 µM on seed weight and chemical constituents in the dry seeds of red kidney beans (*Phaseolus vulgaris* L.) plants at 105 days from sowing. The data are the mean of three replicates. Statistical analysis was carried out using Duncan. Different letters show significant variation at 0.05 P. Vertical bars represent ± SE.

Data presented in Figs. 6 and 7 show that application of Na_2_SeO_4_, GA-SeNPs or Na_2_SO_4_ up to 10 µM, as well as the interaction of sulfur with either Se or nano- Se up to 5 µM, significantly increased the yield expressed as a 100-seed weight (g), total carbohydrate (TC) and crude protein (CP) in the dry seeds of red kidney bean more than control at 105 DAS. While, a reverse situation was observed at 50 µM of Na_2_SeO_4_ and Na_2_SO_4_ in comparison with untreated control plants. The highest increase in 100-seed weight was recorded by applying SeNPs + Na_2_SO_4_, each at 5 µM, followed by SeNPs at 1 µM.

Moreover, SeNPs at the four used concentrations was more effective than Na_2_SeO_4_ and Na_2_SO_4_ in increasing 100-seed weight (100-SW), TC and CP in seeds, SeNPs at 1 µM recorded the maximum increase in 100-SW, TC and CP (increased 67.14, 18.14 and 23.47% more than the control), respectively, followed by Na_2_SO_4_ at 10 µM then Na_2_SeO_4_ at 5 µM concentration. On the other hand, the high concentration (50 µM) of Na_2_SeO_4_ and Na_2_SO_4_ decreased the seed yield and quality compared to control plants (Fig. 7).

Furthermore, the interaction between nano-Se and Na_2_SO_4_ increased SW, TC and CP in red kidney bean seeds more than the combination of Se + Na_2_SO_4_. The greatest increase in 100-SW, TC and CP (increased 70.36, 33.95 and 39.86% more than the control, respectively) were obtained by application of SeNPs at 5 µM + Na_2_SO_4_ at 5 µM, followed by SeNPs + Na_2_SO_4_, each at 2.5 µM (Fig. 7).

### Photosynthetic pigments

**Figure 8:**
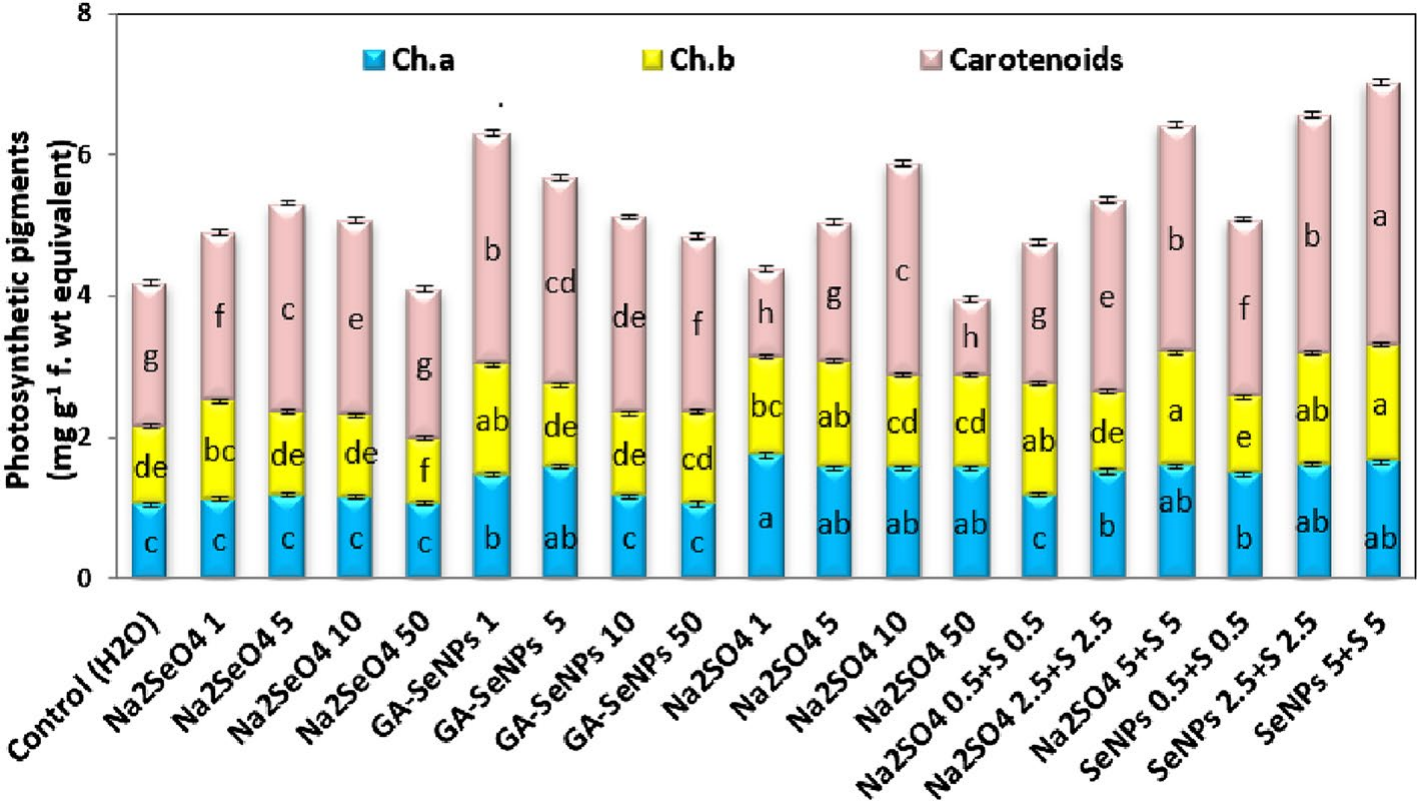
Effect of foliar spray with sodium selenate (Na_2_SeO_4_), selenium nanoparticles (GA-SeNPs), and sodium sulphate (Na_2_SO_4_), each at 0.0, 1, 5, 10 and 50 µM and interaction between sodium sulphate with either sodium selenate or selenium nanoparticles, each at 0.5, 2.5 and 5 µM on photosynthetic pigments contents (mg g^−1^ fresh weight) in the leaves of red kidney bean (*Phaseolus vulgaris* L.) plants at 75 days from sowing. Each result is a mean of 3 replicates. Statistical analysis was carried out using Duncan test. Different letters show significant variation at 0.05 P. Vertical bars represent ± SE.

Foliar spray of red kidney bean plants with Se, nano-Se or Na_2_SO_4_ up to 10 µM as well as the interaction between Na_2_SO_4_ with either Se or nano-Se significantly increased chl a, b, carotenoids and the total photosynthetic pigments (TPP) in the leaves of red kidney bean plants, relative to their corresponding controls at 45 DAS. The most effective concentrations were GA-SeNPs at 1µM, Na_2_SeO_4_ at 5 µM and Na_2_SO_4_ at 10 µM, and their combination at 5 µM (Fig. 8).

Nano-selenium at 1–50 µM concentrations, significantly increased photosynthetic pigments compared to untreated plants. The greatest recorded values of Chl a and b, carotenoids and TPP in the leaves of kidney bean plants were obtained with 1 µM GA-SeNPs, whereas Na_2_SeO_4_ and Na_2_SO_4_ at 50 µM markedly decreased these pigments (Fig. 8).

Moreover, the interaction between GA-SeNPs and Na_2_SO_4_ (0.5–5 µM) was more effective than Na_2_SeO_4_ + Na_2_SO_4_ in increasing the TPP content in the leaves of kidney bean plants at three used concentrations. Nano-Se at 5 µM + sodium sulfate (S) at 5 µM, followed by nano-Se at 2.5 µM + S at 2.5 µM increased TPP by 67.94 and 56.94%, respectively more than control plants (Fig. 8).

### Mineral contents

Data presented in Table 1 show that foliar application of Na_2_SeO_4_, GA-SeNPs or Na_2_SO_4_ up to 10 µM concentration, as well as the combination of Na_2_SO_4_ with either Se or nano-Se increased N%, P, K, Mg, S and Se contents (ppm) in the leaves at 105 DAS as well as N, P, S and Se in the seeds at 75 DAS more than controls. Regarding P levels in seeds, plants from Na_2_SO_4_, Na_2_SeO_4_ and interaction treatments showed slight differences amongst each other, except the plants treated with GA-SeNPs. On the other hand, a slight decrease was observed in the level of N, P and Mg in leaves and similar changes in N and P levels in seeds at 50 µM Na_2_SO_4_ and Na_2_SeO_4_, in comparison with untreated control plants.

**Table 1:** Effect of foliar spray with sodium selenate (Na_2_SeO_4_), selenium nanoparticles (GA-SeNPs), and sodium sulphate (Na_2_SO_4_), each at 0.0, 1, 5, 10, and 50 µM and interaction between sodium sulphate with either sodium selenate or selenium nanoparticles, each at 0.5, 2.5 and 5 µM on leaf and seed mineral concentrations of red kidney bean (*Phaseolus vulgaris* L.) plants.

Treatments (µM)	Mineral contents in leaves (g kg^−1^) × 10^–3^						Mineral contents in seeds (g kg^−1^) × 10^–3^					
	N%	P	K	Mg	S	Se	N%	P	K	Mg	S	Se
	mg kg^−1^	(g kg^−1^) × 10^–3^					mg kg^−1^	(g kg^−1^) × 10^–3^				
Control (H_2_O)	18.3^i^	25.17^b^	25.25^a^	4.57^abc^	1.42^e^	0.025^fg^	27.5^h^	2.35^a^	9.06^ab^	1.66^a^	0.22^d^	0.025^defg^
Na_2_SeO_4_ 1	19.9^fg^	40.83^b^	32.09^a^	4.68^abc^	2.56^bcde^	0.040^bcdefg^	29.9^fg^	2.57^a^	8.93^ab^	1.71^a^	0.31^cd^	0.021^fg^
Na_2_SeO_4_ 5	21.4^de^	41.24^b^	34.40^a^	5.25^abc^	3.08^abcde^	0.043^abcdef^	32.1^de^	2.70^a^	8.32^abc^	1.59^a^	1.03^abcd^	0.024^defg^
Na_2_SeO_4_ 10	2.04^efg^	40.17^b^	31.37^a^	4.68^abc^	3.23^abcde^	0.048^abcd^	30.6^ef^	2.43^a^	7.04^cd^	1.51^a^	0.65^bcd^	0.023^efg^
Na_2_SeO_4_ 50	16.5^j^	25.15^b^	30.27^a^	4.50^bc^	3.70^abcd^	0.050^abc^	24.8^i^	2.13^a^	8.73^ab^	1.11^a^	0.31^cd^	0.016^g^
GA-SeNPs 1	22.6^c^	42.24^b^	35.10^a^	6.10^abc^	3.97^abc^	0.049^abcd^	33.9^c^	3.67^a^	9.63^a^	1.75^a^	1.17^abc^	0.028^cdefg^
GA-SeNPs 5	22.1^cd^	41.12^b^	32.36^a^	4.94^abc^	3.07^abcde^	0.051^abc^	33.2^de^	3.58^a^	9.60^a^	1.61^a^	1.18^abc^	0.025^defg^
GA-SeNPs 10	21.9^cd^	41.04^b^	30.15^a^	4.74^abc^	3.45^abcd^	0.052^ab^	32.9^de^	3.23^a^	9.14^ab^	1.56^a^	1.05^abcd^	0.020^fg^
GA-SeNPs 50	20.6^ef^	40.36^b^	25.40^a^	4.62^abc^	4.19^bd^	0.060^a^	30.9^ef^	2.79^a^	8.83^ab^	1.48^a^	0.42^cd^	0.017^fg^
Na_2_SO_4_ 1	21.2^de^	25.62^b^	25.74^a^	5.66^abc^	3.34^abcde^	0.027^efg^	31.8^e^	2.40^a^	9.18^ab^	1.82^a^	0.41^cd^	0.031^cdefg^
Na_2_SO_4_ 5	21.4^de^	25.62^b^	26.17^a^	5.85^abc^	3.40^abcd^	0.028^efg^	32.1^de^	2.40^a^	9.31^ab^	1.92^a^	0.74^bcd^	0.031^cdefg^
Na_2_SO_4_ 10	21.9^cd^	39.80^a^	28.89^a^	6.54^a^	3.83^abcd^	0.032^cdefg^	32.9^de^	2.44^a^	9.41^a^	2.00^a^	1.69^a^	0.032^cdefg^
Na_2_SO_4_ 50	18.3^i^	25.00^b^	25.56^a^	4.30^c^	4.66^a^	0.022^g^	27.5^h^	2.26^a^	9.14^ab^	1.84^a^	0.90^abcd^	0.036^bcdef^
Na_2_SeO_4_ 0.5 + Na_2_SO_4_0.5	18.9^hi^	26.45^b^	26.7^a^	5.08^abc^	1.86^de^	0.026^fg^	28.4^gh^	2.48^a^	8.71^ab^	1.78^a^	0.40^cd^	0.042^bcde^
Na_2_SeO_4_ 2.5 + Na_2_SO_4_ 2.5	21.2^de^	26.74^b^	27.65^a^	5.54^abc^	2.11^cde^	0.030^defg^	31.8^e^	2.50^a^	7.87^bc^	1.38^a^	0.46^bcd^	0.047^abc^
Na_2_SeO_4_5 + Na_2_SO_4_ 5	24.2^b^	26.87^b^	28.02^a^	6.27^ab^	2.29^bcde^	0.042^abcdef^	32.6^de^	2.57^a^	5.84^d^	1.26^a^	0.56^bcd^	0.051^ab^
GA-SeNPs 0.5 + Na_2_SO_4_ 0.5	1.96^gh^	27.51^b^	27.01^a^	5.91^abc^	2.24^cde^	0.033^bcdefg^	29.4^fg^	2.50^a^	8.96^ab^	1.67^a^	0.60^bcd^	0.043^bcd^
GA-SeNPs 2.5 + Na_2_SO_4_ 2.5	2.17^cd^	26.87^b^	28.02^a^	5.95^abc^	2.49^bcde^	0.035^bcdefg^	36.3^b^	2.52^a^	8.85^ab^	1.45^a^	1.36^ab^	0.047^abc^
GA-SeNPs 5 + Na_2_SO_4_ 5	25.6^a^	30.05^b^	30.38^a^	6.38^ab^	2.55^bcde^	0.046^abcde^	38.4^a^	2.78^a^	8.28^abc^	1.31^a^	1.57^a^	0.062^a^
L.S.D 0.05	1	0.12	NS	0.0017	0.002	0.17	1.7	0.002	0.001	0.0008	0.0008	0.17

Statistical analysis was carried out using Duncan. Different letters show significant variation at 0.05 P.

Generally, GA-SeNPs (1–50 µM) were more effective than Na_2_SeO_4_ and Na_2_SO_4_ in increasing N, P, K and Mg, S and Se contents in leaves, N and P in seeds, while decreased K and Mg in seeds at high concentrations. Plants treated with 1 µM SeNPs showed maximal contents of nitrogen, phosphorus, potassium and Mg (22.6, 0.042, 0.035 and 0.0610 g/kg, respectively) in leaf and (33.9, 0.00367, 0.0096 and 0.00175 g/kg, respectively) in seeds, followed by Na_2_SO_4_ at 10 µM concentration in both leaf and seeds, where, plants treated with 10 µM Na_2_SO_4_ exhibited 1.58- and 1.04-fold increase of total P contents in leaf and seed, respectively. Regarding N levels in seeds, plants showed a 1.20-fold increase in total content, compared with the control. Similarly, K levels increased with the exogenous application of Na_2_SO_4_ in both leaf and seeds. A continuous increase of total Se level was also observed in both leaf and seeds with an increased concentration of Na_2_SO_4_ supply and in leaf with the increased dosages of nano-Se and selenate supply. Noticeably, up to 2.73-fold increases in total Se levels were detected in leaves between treatments with 50 μM of GA-SeNPs and Na_2_SO_4_. Also, increasing selenate, nano-Se and Na_2_SO_4_ supply to 10 μM caused continuous increase in the accumulation of S, showing a correlated change with Se content in the leaves, while the Se levels in the seeds decreased when plants were treated with 50 μM Na_2_SeO_4_ and nano-Se (Table 1).

Furthermore, the interaction between Na_2_SO_4_ with either Se or nano-Se up to 5 µM increased N, P, K, Mg, S and Se content in leaf and similarly increased N, P, S and Se in seed, while decreased K and Mg content in seeds, relative to control red kidney bean plants. The combination of Na_2_SO_4_ with nano-Se at 5 µM increased the content of N, P, Se and S in seed by 1.40, 1.18, 7.14 and 2.48-fold, respectively compared with control followed by combination of Na_2_SO_4_ with nano-Se, each at 2.5 µM (Table 1).

## Discussion

The results obtained show that the foliar application of Na2SeO4, GA-SeNP, or Na2SO4 up to 10 µM and the interaction of sodium sulfate with either Se or nano-Se shows a significant increase in (Shoot and root length, fresh and dry weight, number of roots, leaves and total leaf area of red kidney bean plants^−1^), while high concentrations adversely affected growth. (50 μM) Na2SeO4 and Na2SO4 were compared to corresponding untreated control plants.

It is thought that the competition between Se and S for assimilation into amino acids and proteins may account for the toxicity of Se in most plants^54,55^. Stimulation of red kidney bean growth may be related to increased plant growth-promoting factors, cell division, nutrient uptake, improved photosynthesis and increase in sugar content. In lettuce, Se enhances antioxidant activity and promotes plant growth^56^, enhancing photosynthesis, stomatal conductance, carboxylation efficiency and Rubisco content in *Nicotiana tabacum* L. leaves at 6 mg kg^−1^ Na_2_SeO_3_^57^ and increasing the efficacy of a phytohormone 24-epibrassinolide and acting as quasi essential micronutrient and consequently enhanced the growth and photosynthesis at a low level of Na_2_SeO_4_ (10 µM), while higher concentrations (80 µM) induced deleterious effect in *Brassica juncea* plants^58^. Application of Se at 10 ppm resulted in a significant increment in plant height, the number of leaves, and fresh and dry weights of the stem in faba bean plants^59^. Also, the low level of sodium selenite (2 mg L^−1^) improved shoot and root dry matter production in cucumber plants^60^ and seven wheat (*Triticum aestivum*) lines, at ∼ 5 μM of both selenate and selenite^61^. The present results indicated that the treatment of red kidney bean plants with Na_2_SO_4_ up to 10 µM, as well as the interaction between sodium sulfate with either Se or nano-Se significantly increased all measured growth criteria of kidney bean plants. Our results are in line with Orman and Kaplan^62^, who reported that S application increased the biomass of tomato plants grown in sandy loam soil by 6–8%. The average shoot dry mass of tomato plants increased by 77% following the application of 100 mg kg^−1^ S as compared to the control^63^. An increased yield was observed with foliar application of sodium selenite at50 g ha^−1^ Se on cowpea plants^64^. Also, adding the appropriate amount of S increases plant height, root length and root and shoot dry weights of Tartary buckwheat seedlings, in the absence or presence of Cd^65^. Sulfur nanoparticle (SNP) improved the growth and photosynthetic parameters of lettuce (*Lactuca sativa*) plants at 1 mg mL^−1^, while, higher concentration (10 mg mL^−1^) exhibited toxicity with reducing plant growth and biomass^66^. Tomato Plants foliar sprayed with 6 ppm sulfur attained maximal biomass accumulation as compared to other S treatments and control, indicating the positive role of S in enhancing plant growth and mitigating the effect of heat stress^67^. On the other hand, Na_2_SeO_4_ and Na_2_SO_4_ at 50 μM concentration decreased plant growth. Similarly, Se at high concentration (25 μM) plays a suppressor role on plant growth variables; decreased plant height, leaf area and dry weight in maize plants^68^. This agree with^64^, who stated that application of sodium selenite at high concentrations 1200 and 1600 g ha^−1^ caused leaf toxicity. Increased lipid peroxidation and hydrogen peroxide concentration and reduced total sugars, sucrose, and carotenoid concentration were observed at highest. The toxicity of high Se content was discussed by Hawrylak-Nowak et al.^33^ report in cucumber plants, high concentrations of Se catalyze the oxidation of thiols and their pro-oxidative ability to generate superoxide and damage, resulting in metal toxicity such as growth inhibition, plant height, and root and shoot weight reduction. Symptoms were observed. Cellular component^32^, replacement of S atoms by Se in S-containing amino acids. This leads to protein misfolding, resulting in protein and enzyme dysfunction and reduced plant growth^69^. However, a growing body of research suggests that excess sulfur in crops is a double-edged sword. A low S content promotes plant growth, whereas a high S content inhibits nitrogen uptake and reduces plant production^70^. High SNP concentrations (10 mg mL^−1^) were toxic by inducing oxidative stress markers (H_2_O_2_ and MDA), resulting in decreased lettuce plant growth and biomass^66^. Therefore, proper S content is very important to maintain regular growth of crops. Moreover, in this study, single SeNPs up to 50 μM promoted the growth of red kidney bean plants much better than Na_2_SeO_4_ and Na_2_SO_4_. In this regard, Nano-Se is more efficient in upregulating selenoenzymes^32^.

A high concentration of nano-Se significantly stimulated the organogenesis and the growth of root system in tobacco callus cultures, which was completely inhibited by selenate^34^. According to Hartikainen et al.^71^, the improvement in ryegrass plant growth is believed to be due to the effect of SeNPs on preventing the initiation of growth promoter biosynthesis and/or its disruption. Alternatively, it may be due to a synergistic effect on the stimulatory action of the promoter by converting the inactive form to its promoter. It is the active form and causes changes and stimulation of endogenous growth-promoting hormones. Low concentrations of Se (2.5 μM) and nano-Se (1 μM) improved tomato growth parameters more effectively than high concentrations of Se/nano-Se under high and low temperature stress^36^, Improved the growth parameters of cowpea plants at 6 °C. 25 μM under normal and salt stress^37^.

Our results showed that both low concentrations of selenium and sulfur (Na2SO4) had significant effects on the metabolic activity of red kidney bean plants, which was reflected in improved plant growth standards. Intuitively, the combination of particularly nanoform selenium and low concentrations (0.5–5 μM) of sulfur increased the growth and dry matter production of red kidney bean plants more than single treatments with Se or S.

In the present study, the application of up to 10 μM Na2SeO4, GA-SeNP, or Na2SO4 and the interaction of up to 5 μM Na2SO4 with either Se or Nano-Se significantly increased the yield expressed by 100 seed weights (g), total carbohydrates (TC) and crude protein (CP) in dried kidney bean seeds were higher than controls at 105 DAS, probably due to growth-promoting hormones, photosynthesis, enzymatic activity, and biological effects on translocation processes. Due to regulatory effects, leaves interfere with other plant metabolites and seeds that bind or change to seeds, affecting yield (Fig. 7). In this context, foliar application of sodium selenate significantly increased lentil yield^72^ and affected nutrient uptake, maintenance of turgor pressure, gas exchange properties, and wheat plants. Improved grain yield and quality by increasing the activity of the antioxidant system of Under normal and water-deficient conditions^30^, well-irrigated and dry conditions, soluble carbohydrate and protein levels in leaves and roots of two wheat genotypes increased^73^. The supply of S to plants is essential for vegetative growth and allows production of seeds with high quality. In tomato plants, foliar application of sulfur not only sustained leaf nitrogen, phosphorus, potassium, and proline contents but increased their nutrition levels under heat stress^67^. On the other hand, the high concentration (50 µM) of both Na_2_SeO_4_ and Na_2_SO_4_ markedly decreased the yield and quality of seeds by decreasing (100-SW), TC and CP in comparison with untreated controls plants. In this respect, Se at high concentration (3 μg L^−1^) slightly reduced seed weight (g plant^−1^) and weight of 1000 seeds (g) in two wheat genotypes^73^, minimum carbohydrate content in canola (*Brassica napus* L.) leaves at high Se dosage (10 mg L^−1^)^29^, while nitrogen concentration was not significantly affected by selenate in alfalfa (*Medicago sativa* L.)^74^ and remained at the control level at low selenite (2, 6 µM), but significantly decreased under a highly phytotoxic selenite concentrations (30 and 60 µM) in the aboveground organs in cucumber^33^. Also, high S content restrains nitrogen uptake, which reduces crop production, while low S content facilitates crop growth^70^. The highest increase in 100-SW, TC and CP (increased 67.14, 18.14 and 23.47% more than the control), respectively were obtained at 1 µM GA-SeNPs, followed by Na_2_SO_4_ at 10 µM then Na_2_SeO_4_ at 5 µM concentration. Se-NPs at 400 mg improved yield performance and protein content than at 500 mg fertilization in cluster bean^35^, significantly increased the seed yield in sorghum under high-temperature stress^75^ and increased 100-SW, TC and CP in cowpea seeds at 6.25 µM SeNPs and Na_2_SeO_4_^31^.

Similarly, in this study, the combination of nano-Se and up to 5 μM sodium sulfate provided a highly significant physiological linkage, reflected in a significant improvement in kidney bean yield compared to Se or S alone, it was done. The largest increases in 100-SW, TC, and CP (70.36, 33.95, and 39.86% increases over controls, respectively) were due to interactions between 5 μM nano-Se + 5 μM Na_2_SeO_4_, followed by GA-SeNPs + Na_2_SO_4_, obtained at 2.5 μM, respectively (Fig. 7). This agree with Silva et al.^76^ results on cowpea plants who reported that interaction between 25 g Se ha^−1^ and 30 kg S ha^−1^ was associated with greater sucrose, amino acids, and storage proteins concentrations in cowpea seeds as Selenium uptake and assimilation might be affected by S and vice versa which affects different metabolic pathways such as biosynthesis of sugars, amino acids, and storage proteins. Abdalla et al.^77^ reported that Se and S exhibited a unique synergistic positive effect in increasing amino acids and soluble sugars (glucose, fructose, and sucrose) content in lettuce plants under Se and S enrichment compared to control plants either with limited Sulphur or limited Selenium supply.

Application of Na_2_SeO_4_, GA-SeNPs, or Na_2_SO_4_ up to 10 M, as well as the combination of sulfur with either Se or nano-Se, up to 5 M, significantly increased photosynthetic pigments in red kidney bean leaves, while Na_2_SeO_4_ and Na_2_SO_4_ at 50 M markedly decreased photosynthetic pigments in comparison to untreated control plants. By preserving and preventing chloroplasts from senescing, delaying Chl breakdown, and/or increasing Chl biosynthesis, Na_2_SeO_4_, GA-SeNPs, and Na_2_SO_4_ may simultaneously boost CO_2_ assimilation and photosynthetic rate at low concentrations. In this regard, sodium selenate at 50 mg m^−2^ boosted chlorophyll content throughout the early phases of lettuce plant development, whereas at 100 and 200 mg m^−2^, it prevented senescence and delayed the reduction in total chlorophyll content^78^. In barley, the high selenate dosage had a harmful effect on photosynthesis via changes in activity and/or biosynthesis of enzymes, rather than via alteration of PSII, which is interrelated with the photosynthetic capacity^79^. Chlorophyll b in spinach plants was more responsive to the Se stress than chlorophyll a according to Saffaryazdi et al.^80^. In comparison to the Cd treatment alone, adding 100 mM SO_4_^2−^ improved net photosynthesis in Tartary buckwheat seedlings under Cd stress by 81.60%^65^. SNP at 1 mg mL^−1^ enhanced photosynthetic function in lettuce (*Lactuca sativa*) plants, making them more resilient to harsh situations. According to Najafi et al.^66^, SNP at a dosage of 10 mg mL^−1^ showed harmful effects on all physiological indices. By applying foliar S at 6 ppm to the “Roma” cultivar cultivated at 25 °C, sufficient S nutrition to the plants enhances photosynthesis by boosting chlorophyll creation^81^, maximal CO_2_ index, photosynthetic rate, transpiration rate, and greenness index values. In our study, nano selenium singly was more effective in increasing total photosynthetic pigments in red kidney bean leaves. Similarly, the application of nano-Se at 1 μM improved the chlorophyll content by 27.5% while Na_2_SeO_4_ at 2.5 μM increased it by 19.2% in tomato leaves, under low-temperature stress^36^ and cowpea at 6.5 μM of either nano-Se or Se^31^. Selenium nanoparticles significantly increased total chlorophyll and carotenoid*s* in cluster bean (*Cyamopsis tetragonoloba*) at 400 mg concentration^35^. Similarly, in this study, the combination of nano-Se at 5 µM + S at 5 µM was the treatment that most increased the photosynthetic pigments of red kidney bean leaves.

In the current study, the foliar application of Na_2_SeO_4_, SeNPs, and Na_2_SeO_4_ up to 10 M concentration, as well as the combination of sulfur with either Se or nano-Se, increased the content of N%, P, K, Mg, S, and Se (ppm) in the leaves and N, P, S, and Se in the seeds that the plants produced. The improvement of elements in leaves and seeds as a result of applied Se, NSe, and S-mediated increases in root length and proliferation that boost nutrient uptake from the soil. In this regard, red clover diet, barley grain and straw all showed higher Se levels thanks to treatment with 10 and 20 g Se ha^−1^ of sodium selenate^82^. Se at 5 mg L^−1^ showed a positive effect on P^3+^ and Mg^2+^ levels in rapeseed plants^29^, and a Na_2_SeO_4_ content of 3 μg L^−1^ reduced P, K, and Ca levels increased^73^. However, application of selenate at doses of 2–60 μM increased the phosphorus content in a dose-independent manner and resulted in slightly higher Ca concentrations, whereas increasing selenate concentrations in the growth medium resulted in K value has decreased significantly. With increasing S-SO_4_^−2^, accumulation in cucumber sprouts exceeded 6 μM, making the effect of SeO^−^_4_ ion as a SO_4_^−2^ ion analogue more pronounced^33^. Furthermore, sulfur application significantly increased nitrogen uptake in wheat^83^, improved plant uptake of phosphorus, sulfur, calcium, magnesium and iron, and reduced toxic effects in barley with exogenous NaHS application. Reduced accumulation of aluminum and MDA^84^. Sulfur maximizes nitrogen, phosphorus and potassium uptake and promotes plant growth^63^. In red kidney beans, exogenous application of Na_2_SO_4_ increased K values in both leaves and seeds. The researchers found a positive correlation between K and S contents in shoots, interacting with K as a counteraction for SO_4_^−2^ during vacuolar storage and xylem loading on leaf tissue, elucidated the role^85^. Foliar application of sulfur not only maintained leaf nitrogen, phosphorus, and potassium contents, but also increased leaf nutrient levels in tomato plants under heat stress^67^. In red kidney bean plants, application of Na2SeO4 and Na2SO4 at a concentration of 50 μM decreased leaf N, P and Mg levels and caused similar changes in seed N and P levels, but K and S in leaves increased. In this regard, a competitive relationship is commonly observed between selenate and sulfate uptake in plants when high doses of selenate or sulfate are used^86^. Se can replace S indiscriminately and incorporate Se amino acids in to proteins. The formation of Se-amino acids, in turn, is found to enhance ethylene production which can modify membrane lipid composition^87^, increase membrane permeability and result in an increased K^+^ leakage^88^. Increased K^+^ leakage was caused by high Se addition, and more water was held in the intercellular space to balance out the increased osmotic pressure. Low S content promotes crop growth and high S content inhibits nitrogen uptake, which lowers agricultural yield^70^. In our investigation, a constant rise in total Se levels was seen in seeds and leaf, and this rise was associated with higher sulfur supply concentrations and, for leaf, higher doses of selenate and nano-Se. Also, increasing selenate, nano-Se and sulfur supply to 10 μM caused continuously increased accumulation of S, showing a correlated change with Se content in the leaves (Fig. 8). These results might be partially supported by those of other workers who revealed that selenate at 15 μM enhanced the S level in aboveground plant organs of lettuce^56^ and Se content and S level up to 3.4-fold increase of total S content in shoots of seven wheat (*Triticum aestivum*) lines, compared with control by increasing Na_2_SeO_4_ supply to 10 μM, where selenate-promoted expression of a number of sulfate transporters, resulting in the selenate-induced S accumulation. Also, the increased APS1 and APR2 protein abundance in the selenate-treated leaves might suggest an enhanced S/Se metabolism following the selenate-regulated increase of S level in the leaves of wheat lines^61^. In this study, selenate and nano-Se treatments promoted S accumulation in the leaf, while the S level in seeds decreased, especially at 50 μM. In this respect, while the selenate application at 5 μM was effective in enhancing grain Se level, enhanced S content in both shoots and roots in the wheat lines, the S level in grains, decreased slightly due to a competition between S and Se to translocate into this organ^61^.

Moreover, SeNPs (1–50 µM) was more effective than Na_2_SeO_4_ and Na_2_SO_4_ in increasing N, P, K, Mg S and Se contents in leaf, N and P in seeds, while decreased K and Mg in seeds at high concentrations. Plants treated with 1 µM NSe showed maximal contents of nitrogen, phosphorus, potassium and Mg in leaf and seeds, followed by Na_2_SO_4_ at 10 µM concentration in both leaf and seeds, which might be attributed to the better proliferation and absorption of root by SeNPs application than the Na_2_SeO_4_. Se-NPs increased Se concentrations to 1.7 and 3.4 μg g^−1^ at 50 and 100 mg L^−1^, respectively compared with < 0.05 μg g^−1^ Se for the control sorghum leaf^75^, had a significant effect on N, P, K and Se in both leaves and seeds of pea plants^89^ and cowpea seeds^31^.

Similarly, in this study, the interaction between sodium sulfate with either Se or nano-Se up to 5 µM increased N, P, K, Mg, S and Se content in leaf and N, P, S and Se levels in seed. The greatest increase in the content of N, P, Se and S in red kidney bean seed was obtained by the combination of sodium sulfate with nano-Se, each at 5 and 2.5 µM, indicating their physiological cooperation and reflected in the nutrient improvement in seeds compared to either Na_2_SO_4_, GA_-_ SeNPs or Na_2_SeO_4_, alone.

## Conclusions

In the present work, eco-friendly synthesis of SeNPs was successfully performed through the use of ascorbic acid and gum arabic as natural reducing and stabilizing agents. Foliar application of different concentrations of Na_2_SeO_4_, nano-Se, Na_2_SO_4_ and their interaction can be used to promote vegetative growth and yield of red kidney bean plants at low dosage to avoid selenium accumulation in plant. Selenium and Sulphur coordinating together are more efficient than in separate. Overall, 5 μM nano-Se + 5 μM Na_2_SO_4_ may be used as an effective exogenous application strategy to improve the physiological responses, growth and seed quality of red kidney bean plants. However, further researches are needed to elucidate the interacting role of Se and S in plants from more specific physiological and genetical view.

## Data Availability

All data generated or analyzed during this study are included in this article. The datasets used and/or analysed during the current study available from the corresponding author on reasonable request.
